# Perceived discrimination, health and wellbeing among middle-aged and older lesbian, gay and bisexual people: A prospective study

**DOI:** 10.1371/journal.pone.0216497

**Published:** 2019-05-10

**Authors:** Sarah E. Jackson, Ruth A. Hackett, Igor Grabovac, Lee Smith, Andrew Steptoe

**Affiliations:** 1 Department of Behavioural Science and Health, University College London, London, United Kingdom; 2 Department of Social and Preventive Medicine, Center for Public Health, Medical University of Vienna, Vienna, Austria; 3 Cambridge Centre for Sport and Exercise Sciences, Anglia Ruskin University, Cambridge, United Kingdom; Nathan S Kline Institute, UNITED STATES

## Abstract

**Objective:**

To examine cross-sectional and prospective associations between perceived discrimination in daily life (based on a range of attributes), sexual orientation discrimination, and health and wellbeing in middle-aged and older lesbian, gay and bisexual (LGB) people.

**Methods:**

Data were from 304 LGB men and women aged 41–85 years participating in the English Longitudinal Study of Ageing. Perceived discrimination in daily life was reported in 2010/11. Participants could attribute their discrimination experience to characteristics including age, sex, race, physical disability, and sexual orientation. Self-rated health, limiting long-standing illness, depressive symptoms, quality of life, life satisfaction and loneliness were assessed in 2010/11 and 2016/17. Analyses adjusted for age, sex, ethnicity, partnership status and socioeconomic position.

**Results:**

Perceived discrimination in daily life was reported by 144 (47.4%) participants. Cross-sectionally, perceived discrimination in daily life was associated with increased odds of depressive symptoms (OR = 2.30, 95% CI 1.02 to 5.21), loneliness (OR = 3.37, 95% CI 1.60 to 7.10) and lower quality of life (*B* = -3.31, 95% CI -5.49 to -1.12). Prospectively, perceived discrimination in daily life was associated with increased odds of loneliness (OR = 3.12, 95% CI 1.08 to 8.99) and lower quality of life (*B* = -2.08, 95% CI -3.85 to -0.31) and life satisfaction (*B* = -1.92, 95% CI -3.44 to -0.39) over six-year follow-up. Effect sizes were consistently larger for participants who attributed experiences of discrimination to their sexual orientation compared with those who attributed experiences of discrimination to other reasons (e.g. age, sex, race).

**Conclusion:**

These results provide cross-sectional and prospective evidence of associations between perceived discrimination in daily life and health and wellbeing outcomes in middle-aged and older LGB adults in England.

## Introduction

Population ageing is a global phenomenon [1]. Older adults form a diverse group, with heterogeneity in age, life stage (working or retired), health status, income and wealth, marital status, and living arrangements [2]. They are also diverse in their sexual orientation, although little research has focused on older lesbian, gay and bisexual (LGB) individuals. In the LGB population, the age-related burden of poor health and wellbeing [3] may be compounded by increased risks associated with their sexual orientation [4]. This study examines the impact of perceived discrimination in daily life (based on a range of attributes) and sexual orientation discrimination on the health and wellbeing of middle-aged and older LGB people in England.

Maintaining health and wellbeing in later life is a public health priority [5,6]. Ageing is associated with increased risk of a wide range of mental and physical health complications [3]. Accumulating evidence indicates that individuals who identify as LGB experience poorer health and wellbeing than their heterosexual counterparts [7]. Studies consistently report increased prevalence of enduring psychological or emotional problems among LGB individuals, including psychological distress, depression, anxiety disorders and substance abuse [7–9]. Notably, disparities related to psychological distress are most pronounced among those over the age of 55 [9]. Deficits in physical health have also been documented, with LGB individuals showing increased incidence of long-standing conditions that limit their daily activities (e.g. musculoskeletal problems, arthritis, chronic fatigue syndrome) relative to heterosexuals [10], and rating their general health as worse [4,11].

Discrimination has been identified as a potentially important influence on the health and wellbeing of the LGB population [12–15]. A conceptual framework of “minority stress” posits that stigma, prejudice and discrimination create a hostile and stressful social environment that has adverse consequences for health [12]. This framework may apply to other characteristics beyond sexual orientation, acknowledging that individuals may hold multiple marginalised identities. A substantial body of literature has shown that LGB individuals are much more likely than heterosexuals to experience discrimination related to their sexual orientation [16], with many reporting that discrimination interferes with their ability to have a full and productive life [13]. For some LGB people, experiences of individual discrimination occur in discrete lifetime events (e.g. being fired from a job, being prevented from renting or buying a home) and in day-to-day interactions with others who treat them poorly [13]. For others, experiences are the result of institutional discrimination where laws and policies in the public domain perpetuate inequalities (e.g. the prohibition of same-sex marriage), or where laws fail to protect against discrimination based on sexual orientation [10,17]. In England, homosexuality was considered a criminal offence until 1967 [18], so for many older people, much of their sexual development happened under threat of imprisonment. Same-sex partnerships were not legally recognised until the Civil Partnership Act was introduced in 2004 [19]. Older LGB adults in England have therefore lived most of their lives in the context of prevalent institutional discrimination.

The perception of discrimination can be based on multiple attributes such as age, sex, race as well as sexual orientation. Discrimination based on a number of different attributes is perceived to be widespread in Europe [20]. According to a survey of 27,718 individuals, discrimination based on ethnicity was the most frequently reported type of discrimination (64%), followed by sexual orientation (58%), being aged 55 years or older (42%) and gender (37%). Those with multiple marginalised identities may experience even greater levels of discrimination. Discrimination may be particularly harmful among older than younger LGB people given the youth-masculinity culture that is prevalent in the LGBT community [21]. Meta-analytic evidence suggests that gender and ethnicity may influence experiences of victimisation among LGB individuals [16].

An increasing body of work has examined experiences of discrimination in daily life as a determinant of mental health and wellbeing. A 2009 pooled analysis of 110 studies found that perceived discrimination (based on any attribution) was associated with poorer mental health and wellbeing outcomes, including increased depressive symptoms and decreased life satisfaction, quality of life and self-rated health [14]. However, the evidence on the impact of perceived everyday discrimination on the health and wellbeing of LGB individuals is limited [14]. In a study of LGB (*n* = 73) and heterosexual (*n* = 2,844) adults aged 25–74 years (LGB 77.5% <45 years, heterosexual 53.7% <45 years), LGB adults had more frequent experiences with discrimination and 42% attributed this discrimination to their sexual orientation. Perceived (non-attribute specific) discrimination was associated with higher prevalence of mental health disorders, greater psychological distress and poorer self-rated mental health in the whole sample [13]. In another survey of LGB and transgender (LGBT, *n* = 472, mean age 38.9 years) and heterosexual (*n* = 7,412, mean age 40.0 years) adults, recent experience of a major incident of discrimination attributed to any characteristic was associated with a range of indices of mental health and lower utilisation of mental health care services [22]. Among the LGB and transgender participants, just over half (51%) attributed the discrimination incident to their sexual orientation. The existing evidence base lacks prospective data on the impact of discrimination in daily life on health and wellbeing among LGB individuals. In addition, no studies to our knowledge have investigated this (either cross-sectionally or prospectively) among older adults. Furthermore, it is unclear whether the attribution of the discrimination to sexual orientation in particular has an influence on the relationship between perceived discrimination and health and wellbeing outcomes.

This study therefore aimed to examine associations between perceived discrimination in daily life and health and wellbeing in a sample of middle-aged and older LGB people drawn from a nationally-representative survey of middle-aged and older adults in England. Specifically, we analysed cross-sectional and prospective associations between perceived discrimination and self-rated health, limiting long-standing illness, depressive symptoms, life satisfaction, quality of life and loneliness using data collected over a period of six years. These variables were selected in order to provide a comprehensive picture of health and wellbeing, encompassing general health, the presence of chronic conditions, positive and negative affect, global wellbeing, and social wellbeing. We also assessed whether the pattern of results remained the same for those who perceived discrimination but did not attribute it to their sexual orientation and those who perceived discrimination on the basis of their sexual orientation.

## Method

### Study population

Data were from the English Longitudinal Study of Ageing (ELSA), a longitudinal panel study of middle-aged and older men and women living in households in England [23]. ELSA participants are drawn from households with one or more members 50 years or older responding to the Health Survey for England (HSE). All household members 50 years or older plus partners who are younger than 50 years or had joined the household since the HSE are invited for interview. The study began in 2002, with data collected at two-year intervals via computer-assisted personal interview and self-completion questionnaires. The present analyses use data from Wave 5 (2010/11; the only wave in which discrimination has been assessed) and Wave 8 (2016/17; the most recent wave for which data were available). Information on sexual orientation was collected in Wave 6 (2012/13) and used to identify LGB participants for inclusion in our sample. Of those who reported their sexual orientation (*n* = 6,939), 406 (5.9%) were identified as LGB. We would expect sexual orientation to be a stable characteristic in this age group, so would not expect it to differ between Waves 5 and 6. We restricted our sample to LGB participants who completed the assessment of discrimination in Wave 5. This left a final sample for analysis of 304 men and women for cross-sectional analyses, of whom 254 (83.6%) provided follow-up data in Wave 8 and were therefore included in prospective analyses. Ethical approval was obtained from the London Multi-Centre Research Ethics Committee. All participants gave full informed written consent.

### Measures

#### Sexual orientation

Sexual orientation was assessed with the question: “*Which statement best describes your sexual desires over your lifetime*? *Please include being interested in sex*, *fantasising about sex or wanting to have sex*”. Response options were 1) entirely for women, 2) mostly for women, but some desires for men, 3) equally for women and men, 4) mostly for men, but some desires for women, 5) entirely for men, and 6) no sexual desires in lifetime. We categorised participants with desires entirely for the opposite sex as heterosexual, entirely for the same sex as gay and those endorsing response options 2, 3 or 4 as bisexual. We coded the sexual orientation of those reporting no sexual desires as missing. No data were collected on gender identity, so we were unable to include a group of gender non-conforming people. Due to the low numbers of LGB participants in our sample, we analysed data using a binary sexual orientation variable (heterosexual vs. LGB).

#### Perceived discrimination

Items on discrimination were based on measures used in other studies [24–26]. Participants were asked about the frequency of five discriminatory experiences: “*(1) you are treated with less respect or courtesy; (2) you receive poorer service than other people in restaurants and stores; (3) people act as if they think you are not clever; (4) you are threatened or harassed; (5) you receive poorer service or treatment than other people from doctors or hospitals (almost every day/at least once a week/a few times a month/a few times a year/less than once a year/never)*.” Because data were skewed, with most participants reporting never experiencing discrimination, we dichotomised responses to indicate whether or not participants had experienced discrimination in the past year (a few times or more a year vs. less than once a year or never), with the exception of the fifth item which was dichotomised to indicate whether or not respondents had ever experienced discrimination from doctors or hospitals (never vs. all other options) [27].

A follow‐up question asked participants who reported discrimination in any of the situations to indicate the reason(s) they attributed to their experience from a list of options including age, sex, race, physical disability, and sexual orientation. Participants could attribute more than one reason to their experiences of discrimination.

#### Health and wellbeing

Depressive symptoms were assessed with an eight-item version of the Center for Epidemiologic Studies Depression Scale (CES-D) [28]. Respondents were asked to indicate if they had experienced depressive symptoms (e.g. restless sleep and being unhappy) over the past month using a binary (yes/no) response. Total scores ranged from 0 to 8 with higher scores indicating more depressive symptoms. Data were dichotomised using an established cut‐off, with a score of 4 or higher indicating significant symptomatology [29].

Loneliness was measured using the three-item Revised UCLA Loneliness Scale [30]. Participants were asked to rate items including: “*How often do you feel you lack companionship*?” (hardly ever or never = 1 to often = 3). Scores ranged from 3 to 9, with higher scores indicating greater loneliness. They were dichotomised at ≥6 versus <6 to indicate high vs. low loneliness [31].

Quality of life was assessed with the CASP-19 [32], a scale designed to measure quality of life in older people. Items cover several domains of quality of life including control, autonomy, self-realisation and pleasure. Respondents are asked how often each statement applies to them (often = 0 to never = 3). Positively-worded items were reverse scored. A higher score indicates higher quality of life (range: 0–57).

Life satisfaction was assessed with the Satisfaction With Life Scale [33], which asks respondents to rate the extent to which they agree with five statements (e.g. “*In most ways my life is close to my ideal”*) on a scale from 0 (strongly disagree) to 6 (strongly agree). Responses were summed to produce a total score (range: 0–30), with higher scores indicating greater life satisfaction.

Self‐rated health was assessed using a single item: *“Would you say your health is… poor/fair/good/very good/excellent*?*”* We analysed the proportion of individuals rating their health as fair/poor, as has been done in other investigations [34,35].

Limiting long-standing illness was assessed with two questions: (1) “*Do you have any long-standing illness*, *disability*, *or infirmity*? *By long-standing I mean anything that has troubled you over a period of time or that is likely to affect you over a period of time*.*”* If they responded yes, they were asked (2) *“Does this illness or disability limit your activities in any way*?*”* Affirmation of a long-standing illness and any form of limitation classified the participant as having a limiting long-standing illness.

#### Covariates

We included information on age, sex, ethnicity (white vs. non-white), partnership status (married/cohabiting vs. neither) and household non-pension wealth (a sensitive indicator of socioeconomic status in this population [36]).

### Statistical analysis

Analyses were conducted using SPSS version 24. Data were weighted to correct for sampling probabilities and for differential non-response and to calibrate back to the 2011 National Census population distributions for age and sex.

In order to maximise the available sample for analysis, we imputed missing values for all covariates. A multiple imputation model was run with age, sex, ethnicity, partnership status and wealth entered as predictors. A total of 40 missing values were imputed for wealth and 1 missing value was imputed for ethnicity. Five imputed data sets were created, each was analysed separately, and the results were combined to produce pooled estimates of effects; allowing the analyses to account for uncertainty caused by estimating missing data. Pooled estimates are reported throughout the paper.

Bivariate associations between perceived discrimination and covariates (assessed at baseline) were tested using independent-samples t-tests for continuous variables and χ^2^ tests for categorical variables. For our primary analyses, we examined differences in depressive symptoms, loneliness, quality of life, life satisfaction, self-rated health and limiting long-standing illness between those who reported perceived discrimination and those who did not, both cross-sectionally and prospectively over six-year follow-up. Categorical outcomes were analysed using logistic regression and continuous outcomes were analysed using linear regression. We ran two sets of models: the first was unadjusted, the second controlled for age, sex, ethnicity, partnership status and wealth (covariates chosen *a priori*). For prospective analyses, the adjusted models also controlled for baseline status/score on the outcome of interest. As there were few differences in the pattern of results, we present the adjusted models and comment on any differences between the unadjusted and adjusted models in the results section. However, the full results of the unadjusted models are available to view in S1 Table. Results are presented as unstandardized B and 95% confidence intervals (CI) for continuous outcomes and odds ratios (ORs) and 95% CI for categorical outcomes.

As a secondary analysis, we repeated models using a three-level discrimination variable to analyse differences in health and wellbeing outcomes between participants who did not perceive discrimination, those who perceived discrimination but did not attribute it to their sexual orientation, and those who perceived discrimination on the basis of their sexual orientation. Categorical outcomes were analysed using logistic regression and continuous outcomes were analysed using one-way independent analysis of variance with planned contrasts. In each model, the group who did not report perceived discrimination was the reference category. As in the primary analyses, all models controlled for socio-demographic covariates and the prospective analyses also adjusted for baseline status/score on the outcome of interest.

## Results

The 304 middle-aged and older LGB adults in our sample included 103 men and 201 women, ranging in age from 41 to 85 years. Perceived discrimination in daily life based on a range of attributes was reported by 144 (47.4%) participants. Some 34.2% reported being treated with less respect or courtesy, 23.7% reported receiving poorer treatment from doctors or hospitals, 19.4% reported people acting as if they were not clever, 15.9% reported receiving poorer service in restaurants or stores, and 9.5% reported being threatened or harassed.

Characteristics of the groups who did and did not report perceived discrimination in daily life are shown in Table 1. On average, participants who reported perceived discrimination in daily life were significantly younger than those who did not (61.3 vs. 65.3 years, *p*<0.001), and a higher proportion were non-white (7.4% vs. 0.7%, *p* = 0.003) and from the lowest quintile of wealth (24.5% vs. 9.0%, *p* = 0.017). A slightly higher proportion of the group who perceived discrimination in daily life than the group who did not was male, but this difference was not statistically significant (44.6% vs. 34.3%, *p* = 0.093). There was no significant association between perceived discrimination in daily life and partnership status (*p* = 0.854) or sexual orientation (i.e. lesbian, gay or bisexual, *p* = 0.334).

**Table 1:** Sample characteristics at baseline in relation to perceived discrimination in daily life (on the basis of a range of attributes).

			Perceived discrimination (*n* = 144)^1^	No perceived discrimination (*n* = 160)	*p*
Age (years), mean (SE)		61.30 (0.52)	65.34 (0.71)	<0.001
Sex				
	Men		44.6	34.3	0.093
	Women		55.4	65.7	-
Ethnicity				
	White	92.6	99.3	0.003
	Non-white	7.4	0.7	-
Partnership status				
	Married/cohabiting	63.3	64.4	0.854
	Other	36.7	35.6	-
Wealth quintile				
	1 (poorest)	24.5	9.0	0.017
	2	16.0	20.9	-
	3	18.3	17.2	-
	4	20.5	24.6	-
	5 (richest)	20.8	28.4	-
Sexual orientation				
	Lesbian	3.3	3.7	0.334
	Gay	6.6	11.9	-
	Bisexual	90.1	84.3	-

^1^ Unweighted sample sizes.

All figures are weighted for sampling probabilities and differential non-response.

Values are percentages unless otherwise stated.

SE = standard deviation.

Cross-sectionally, after adjustment for age, sex, ethnicity, partnership status and wealth, participants who perceived discrimination in daily life had over 2 times higher odds of having depressive symptoms above threshold (OR = 2.30, 95% CI 1.02 to 5.21, *p* = 0.046) and over 3 times higher odds of high loneliness (OR = 3.37, 95% CI 1.60 to 7.10, *p* = 0.001) relative to those who did not perceive discrimination (Table 2). They also had significantly lower quality of life (*B* = -3.31, 95% CI -5.49 to -1.12, *p* = 0.003). Odds of fair/poor self-rated health were almost twice as high in the group who perceived discrimination in daily life as in the group who did not but the difference did not reach statistical significance in the adjusted model (OR = 1.95, 95% CI 0.91 to 4.19, *p* = 0.085; unadjusted OR = 2.14, 95% CI 1.19 to 3.86, *p* = 0.011). Prevalence of limiting long-standing illness was also slightly higher, and mean life satisfaction scores were slightly lower, in the group reporting perceived discrimination in daily life, but differences were not statistically significant in the adjusted models (limiting long-standing illness *p* = 0.211, life satisfaction *p* = 0.271).

**Table 2:** Cross-sectional and prospective associations between perceived discrimination (on a range of attributes) and health and wellbeing outcomes.

		Cross-sectional		Prospective	
		No perceived discrimination	Perceived discrimination	No perceived discrimination	Perceived discrimination
Depressive symptoms above threshold					
	% (SE)	13.3 (3.1)	24.5 (3.3)	12.4 (2.6)	12.8 (2.9)
	OR [95%CI]	1.00 (Ref)	2.30 [1.02; 5.21]*	1.00 (Ref)	1.39 [0.42; 4.66]
High loneliness					
	% (SE)	17.3 (3.3)	34.8 (3.5)	14.8 (3.2)	25.7 (3.5)
	OR [95%CI]	1.00 (Ref)	3.37 [1.60; 7.10]**	1.00 (Ref)	3.12 [1.08; 8.99]*
Quality of life					
	Mean score (SE)	43.15 (0.70)	39.68 (0.76)	43.21 (0.54)	41.15 (0.59)
	Coeff. [95%CI]	Ref	-3.31 [-5.49; -1.12]**	Ref	-2.08 [-3.85; -0.31]*
Life satisfaction					
	Mean score (SE)	20.93 (0.52)	20.00 (0.57)	21.79 (0.46)	19.98 (0.51)
	Coeff. [95%CI]	Ref	-0.92 [-2.55; 0.72]	Ref	-1.92 [-3.44; -0.39]*
Fair/poor self-rated health					
	% (SE)	19.2 (3.2)	28.6 (3.5)	25.9 (3.2)	24.4 (3.5)
	OR [95%CI]	1.00 (Ref)	1.95 [0.91; 4.19]	1.00 (Ref)	1.07 [0.43; 2.64]
Limiting long-standing illness					
	% (SE)	31.0 (3.8)	40.0 (4.1)	33.8 (3.5)	36.1 (3.9)
	OR [95%CI]	1.00 (Ref)	1.49 [0.80; 2.77]	1.00 (Ref)	1.24 [0.55; 2.80]

All figures are weighted for sampling probabilities and differential non-response and adjusted for age, sex, ethnicity, partnership status and wealth. Prospective figures are additionally adjusted for baseline status/score.

SE = standard error, OR = odds ratio, CI = confidence interval, Ref = reference, Coeff = coefficient.

*p<0.05

**p<0.01

***p<0.001.

Possible scores on the quality of life scale range from 0–57, and on life satisfaction scale range from 0–30.

Prospectively, after adjustment for covariates including baseline levels, participants who perceived discrimination on the basis of any of the listed attributions had over 3 times higher odds of high loneliness (OR = 3.12, 95% CI 1.08 to 8.99, *p* = 0.036) and significantly lower mean quality of life (*B* = -2.08, 95% CI -3.85 to -0.31, *p* = 0.021) and life satisfaction (*B* = -1.92, 95% CI -3.44 to -0.39, *p* = 0.014) than those who did not (Table 2). There was no significant association between perceived discrimination in daily life and self-rated health (*p* = 0.889), limiting long-standing illness (*p* = 0.605) or depressive symptoms (*p* = 0.590).

Of the group who reported perceived discrimination (*n* = 144), 12.5% (*n* = 18) attributed their experiences of discrimination to their sexual orientation. In stratified analyses, effect sizes were consistently larger for the group who attributed experiences of perceived discrimination to their sexual orientation than for those who did not (Table 3). In addition to the significant associations identified in the primary analyses, these models revealed a significant cross-sectional association with self-rated health in the group who attributed experiences of discrimination to their sexual orientation (OR = 6.76, 95% CI 1.65 to 27.63, *p* = 0.008). The cross-sectional association with depressive symptoms and the prospective association with loneliness were only statistically significant for the group who attributed experiences of discrimination to their sexual orientation. The cross-sectional association with quality of life and the prospective association with life satisfaction were only statistically significant for the group who did not attribute experiences of discrimination to their sexual orientation.

**Table 3:** Cross-sectional and prospective associations between perceived discrimination attributed to sexual orientation or any other reason and health and wellbeing outcomes.

		Cross-sectional			Prospective		
		No perceived discrimination	Perceived discrimination for any other reason	Perceived discrimination based on sexual orientation	No perceived discrimination	Perceived discrimination for any other reason	Perceived discrimination based on sexual orientation
Depressive symptoms above threshold							
	% (SE)	12.2 (3.1)	22.2 (3.3)	48.4 (9.3)	12.8 (2.7)	12.1 (3.0)	14.0 (8.3)
	OR [95%CI]	1.00 (Ref)	2.12 [0.90; 4.97]	9.32 [2.11; 41.18]**	1.00 (Ref)	1.21 [0.35; 4.19]	1.49 [0.21; 10.78]
High loneliness							
	% (SE)	16.5 (3.4)	33.4 (3.7)	48.3 (10.2)	14.4 (3.2)	23.7 (3.6)	40.0 (9.5)
	OR [95%CI]	1.00 (Ref)	3.39 [1.56; 7.38]**	6.43 [1.55; 26.75]*	1.00 (Ref)	2.62 [0.88; 7.75]	7.43 [2.99; 18.47]*
Quality of life							
	Mean score (SE)	43.20 (0.71)	39.89 (0.78)	38.66 (2.27)	43.24 (0.55)	41.55 (0.61)	38.80 (1.59)
	Coeff. [95%CI]	Ref	-3.31 [-5.46; -1.16]**	-4.54 [-9.27; 0.19]	Ref	-1.69 [-3.36; -0.02]*	-4.44 [-7.79; -1.08]*
Life satisfaction							
	Mean score (SE)	20.94 (0.53)	20.26 (0.59)	18.08 (1.70)	21.77 (0.47)	20.11 (0.53)	19.83 (1.39)
	Coeff. [95%CI]	Ref	-0.69 [-2.29; 0.92]	-2.86 [-6.40; 0.67]	Ref	-1.66 [-3.10; -0.23]*	-1.95 [-4.86; 0.98]
Fair/poor self-rated health							
	% (SE)	18.2 (3.3)	26.4 (3.6)	51.9 (9.9)	26.4 (3.2)	22.4 (3.6)	35.6 (10.1)
	OR [95%CI]	1.00 (Ref)	1.70 [0.77; 3.76]	6.76 [1.65; 27.63]**	1.00 (Ref)	0.88 [0.34; 2.25]	2.10 [0.34; 12.97]
Limiting long-standing illness							
	% (SE)	30.5 (3.9)	39.5 (4.2)	45.6 (11.8)	33.4 (3.6)	34.3 (4.0)	53.5 (11.0)
	OR [95%CI]	1.00 (Ref)	1.45 [0.76; 2.74]	1.64 [0.45; 5.99]	1.00 (Ref)	1.05 [0.47; 2.33]	3.79 [0.75; 19.19]

All figures are weighted for sampling probabilities and differential non-response and adjusted for age, sex, ethnicity, partnership status and wealth. Prospective figures are additionally adjusted for baseline status/score.

SE = standard error, OR = odds ratio, CI = confidence interval, Ref = reference, Coeff = coefficient.

*p<0.05

**p<0.01

***p<0.001.

Possible scores on the quality of life scale range from 0–57, and on life satisfaction scale range from 0–30.

## Discussion

In this population-based sample of middle-aged and older LGB adults in England, perceived discrimination in daily life (based on a range of attributes) was associated with significantly poorer wellbeing. In cross-sectional analyses, participants who reported perceived discrimination in daily life had increased odds of depressive symptoms and loneliness, and poorer quality of life. Longitudinally, perceived discrimination in daily life was associated with increased odds of loneliness, poorer quality of life, and lower life satisfaction over six years. These results were independent of participants’ age, sex, ethnicity, partnership status and socioeconomic position. Effect sizes were consistently larger for participants who attributed experiences of perceived discrimination to their sexual orientation compared with those who attributed experiences of perceived discrimination to other reasons (e.g. age, sex and race).

Associations between perceived discrimination in daily life (based on a range of attributes) and poorer wellbeing are in line with previous research in heterosexual older samples [14,37] and younger LGB samples [13,14,22]. Consistent with evidence that LGB adults who experience discrimination suffer poorer mental health [13,22,38], participants reporting perceived discrimination in daily life in our sample had more than double the odds of depressive symptoms above the threshold for clinical relevance in cross-sectional analyses. However, there was no prospective association between perceived discrimination in daily life and depressive symptoms over six-year follow-up. It is possible that the impact of ongoing discrimination across the lifespan on mood had already become evident by the time of the baseline survey, limiting scope for further deterioration over time, or that the instrument used to measure depressive symptoms was not sensitive to change.

In contrast, we observed significant associations between perceived discrimination in daily life and loneliness in both cross-sectional and prospective models. Middle-aged and older LGB people who reported perceived discrimination (based on a range of attributes) had over three times higher odds of being lonely cross-sectionally and over six-year follow-up. Previous studies have shown that older LGB people tend to be more likely than older heterosexual people to live alone, be childless, and grow old without a life partner or “significant other” [39]. The Civil Partnership Act legalised same-sex unions in 2004, and the Marriage (Same Sex Couples) Act legalised same-sex marriage in 2013, when participants were on average in their 50s and 60s. The inability to form legal partnerships until later in life likely contributed to greater loneliness and poorer wellbeing among older LGB people. Perceived discrimination (based on a range of attributes) may exacerbate loneliness among older LGB people by making them feel less accepted by society and causing them to avoid social situations where they anticipate they may encounter discrimination [12]. Feelings of loneliness among older LGB people may be exacerbated by the prominent youth culture in the LGBT community [21]. The LGBT community is particularly tight-knit [39], and studies have found that LGB individuals who report a greater feeling of belonging to a community of identity also possess an increased sense of social and psychological wellbeing [40]. Older LGB people, who have likely spent much of their lives feeling excluded from society, now live in a world where this side of their lives can be (to a degree) freely expressed. However, with its strong emphasis on youth, they may find themselves rejected by the LGBT community, worsening loneliness and depressive symptoms. Our results also provide prospective evidence of associations between perceived discrimination and lower quality of life and life satisfaction, suggestive of a potential causal role of discrimination in the association between LGB sexual orientation and these experiences.

Interestingly, just one in eight of those who reported perceived discrimination attributed these experiences to their sexual orientation. While this figure may seem surprisingly low, particularly in comparison with the 42% of LGB adults in a previous survey in the USA of 25–74 year-olds (77.5% <45 years) reporting sexual orientation as the basis for experiences of perceived discrimination [13], in the context of the older age of the sample it is understandable. As an internal dimension of diversity, sexual orientation may be more difficult to recognise than other frequently discriminated characteristics, such as race, age or disability [41]. For example, a person might not necessarily reveal their sexual orientation during a medical appointment, but they cannot hide their age. With older people rarely considered to be sexual beings [42], sexual orientation, a key aspect of sexuality, may be even less likely to attract discriminatory behaviour from others than it would in younger LGB individuals.

When we compared associations between perceived discrimination and health and wellbeing outcomes in those who attributed experiences of perceived discrimination to their sexual orientation with those who did not, we observed substantially larger effect sizes for the group who reported perceived discrimination on the basis of their sexual orientation. Compared with those who did not perceive discrimination, middle-aged and older LGB adults who reported perceived discrimination on the basis of their sexual orientation had more than six times higher odds of loneliness and more than nine times higher odds of depressive symptoms in cross-sectional analyses; statistically significant differences despite the very small sample size. The small size of this subgroup and the large confidence intervals mean that these results should be interpreted cautiously. They also had more than six times higher odds of fair/poor self-rated health; a significant difference that was not observed in the adjusted model when the groups who did and did not attribute perceived discriminatory experiences to their sexual orientation were combined. Prospectively, middle-aged and older LGB people who reported perceived discrimination had significantly lower mean quality of life and had more than seven times higher odds of being lonely. These results indicate that perception of discrimination on the basis of sexual orientation was more strongly linked with health and wellbeing outcomes than was discrimination attributed to other characteristics. It may be that group identity–in this case, as a LGB person–has an important influence on the impact of perceived discrimination on health and wellbeing. It is possible that perceived discrimination on the basis of this core aspect of identity may have a more detrimental impact upon health and wellbeing than other personal characteristics, such as age or sex.

These findings have important implications for the health and wellbeing of a substantial proportion of the older population. The best available estimate of the size of the LGB population in England is 2.5% (range 2.5% to 5.89%) [43]. Based on population estimates provided by the Office for National Statistics [44], this equates to approximately 1.13 million LGB adults in England, of whom around 253,000 (up to a maximum of 596,000 based on the upper limit) are over the age of 65. With the number of older adults projected to continue to rise substantially over coming years [44], the number of LGB individuals aged ≥65 looks set to surpass 500,000 (up to a maximum of 1.2 million) by 2041. Initiatives tackling discrimination against LGB people could help to mitigate the increased risk of poor health and wellbeing in this rapidly expanding population group. These initiatives should recognise that individuals might hold multiple marginal identities and as such the primary attribution for discrimination in this group may not be solely based on their sexual orientation. Addressing stigma and discrimination in healthcare settings may be particularly relevant to improving health outcomes in middle aged and older LBG people. Previous literature has noted important differences between LGB and heterosexuals in health insurance coverage, access to healthcare, and use of preventive services [45,46], which may have a direct impact on health. In the present study one in four participants reported receiving poorer treatment from doctors and hospitals. Addressing the issue of LGB bias in medical training, where heterosexism and anti-LGB discrimination occurs frequently [47], may help to promote a more equal health service for future generations.

Strengths of this study include the prospective design and inclusion of a variety of validated measures of health and wellbeing. The majority of previous studies investigating the health impacts of other forms of discrimination have relied on cross-sectional designs and convenience sampling [14]. Additionally, the questions on sexual orientation were drawn from a questionnaire that was based on Britain’s National Survey of Sexual Attitudes and Lifestyles (Natsal) and other studies where reliability and validity have been extensively assessed [48]. However, there were also several limitations. Previous studies have indicated reluctance of LGB people to come out to researchers [4,49], so it is possible that LGB participants were more likely than heterosexual participants to choose not to answer the questions on sexual orientation used to identify the present study sample. There were no data available on whether participants were “out”. Those who are not out are often plagued by internalised homonegativity and self-hatred, which may influence both the perception of discrimination and health and wellbeing [50]. In addition, the measure of sexual orientation asked about same-sex preference over the life course, which may not necessarily equate to LGB identity. Although the sample was drawn from a large, representative sample of middle-aged and older adults in England, the prevalence of LGB sexual orientation was just 2.1% (in the same region as estimates from Natsal [51]) so our sample was small, limiting statistical power to detect significant associations. That we observed a number of statistically significant differences between LGB individuals who did and did not report perceived discrimination (based on a range of attributes) despite the small sample size attests to the strength of these associations. However, the stability of the estimates relating to sexual orientation discrimination is likely to be low given the small sample size. Replication in a larger sample is required to confirm these findings and provide more reliable estimates of effect size. Perceived discrimination was determined by self-reports of past experiences, introducing scope for bias. In addition, because reports of discrimination were subjective, the results estimate the impact of believing that one has been a target of discrimination as opposed to the impact of discrimination per se. The discrimination questions asked about five situations, but there may be others that are relevant to LGB individuals that were not assessed. Prospective analyses were restricted to participants who had data at six-year follow-up. Attrition between data collection waves meant that there were no follow-up data available for 50 of the participants included in the cross-sectional analyses. The analysed sample was slightly younger and wealthier as compared with the total ELSA sample, so results may not be representative.

In conclusion, these results indicate that among middle-aged and older LGB adults in England, perceived discrimination on the basis of any attribution is associated with deficits in a range of measures of health and wellbeing. While significant progress has been made in reducing institutional discrimination through legislation that offers greater equality to sexual minorities and other protected groups, a substantial proportion of LGB people experience discrimination in their everyday lives. The present findings underscore the need for further action to address discrimination against sexual minorities on a societal level in order to address demonstrated disparities in health and wellbeing between LGB and heterosexual people. Given they are already at increased risk of age-associated health complications, older LGB people may be particularly vulnerable to the detrimental impact of perceived discrimination in daily life, particularly as they may hold multiple marginalised identities. Tackling ageism within the LGBT community could further help to improve outcomes for this population group.

## Supporting information

S1 Table Unadjusted cross-sectional and prospective associations between perceived discrimination and health and wellbeing outcomes. (DOCX)
