# Supplementary material for: Perceived discrimination, health and wellbeing among middle-aged and older lesbian, gay and bisexual people: A prospective study
Source: PLoS One. 2019 May 10;14(5):e0216497. doi: 10.1371/journal.pone.0216497 (PMC6510440; doi:10.1371/journal.pone.0216497)
Supplement: S1 Table — (DOCX) [file pone.0216497.s001.docx]

| **Supplementary Table 1** Unadjusted cross-sectional and prospective associations between perceived discrimination and health and wellbeing outcomes | | | | | | | | |
| --- | --- | --- | --- | --- | --- | --- | --- | --- |
|  | |  |  | **Cross-sectional** | |  | **Prospective** | |
|  | |  |  | **No perceived discrimination** | **Perceived discrimination** |  | **No perceived discrimination** | **Perceived discrimination** |
| Fair/poor self-rated health | | | |  |  |  |  |  |
|  | % (SE) | | | 17.2 (3.4) | 30.8 (3.6) |  | 25.3 (3.2) | 25.1 (3.4) |
|  | OR [95%CI] | | | 1.00 (Ref) | 2.14 [1.19; 3.86]* |  | 1.00 (Ref) | 1.06 [0.49; 2.29] |
| Limiting long-standing illness | | | |  |  |  |  |  |
|  | % (SE) | | | 29.8 (3.9) | 41.4 (4.1) |  | 34.9 (3.6) | 34.8 (3.9) |
|  | OR [95%CI] | | | 1.00 (Ref) | 1.72 [1.02; 2.90]* |  | 1.00 (Ref) | 0.98 [0.49; 1.96] |
| Depressive symptoms above threshold | | | |  |  |  |  |  |
|  | % (SE) | | | 10.8 (3.1) | 27.3 (3.3) |  | 12.5 (2.6) | 12.6 (2.9) |
|  | OR [95%CI] | | | 1.00 (Ref) | 3.15 [1.58; 6.26]** |  | 1.00 (Ref) | 1.14 [0.41; 3.17] |
| Quality of life | | | |  |  |  |  |  |
|  | Mean score (SE) | | | 43.36 (0.74) | 39.45 (0.79) |  | 42.97 (0.55) | 41.43 (0.59) |
|  | Coeff. [95%CI] | | | Ref | -3.89 [-6.13; -1.64]** |  | Ref | -1.51 [-3.26; -0.25] |
| Life satisfaction | | | |  |  |  |  |  |
|  | Mean score (SE) | | | 21.11 (0.56) | 19.79 (0.60) |  | 21.76 (0.46) | 20.01 (0.50) |
|  | Coeff. [95%CI] | | | Ref | -1.38 [-3.09; 0.33] |  | Ref | -1.90 [-3.44; -0.36]* |
| High loneliness | | | |  |  |  |  |  |
|  | % (SE) | | | 15.3 (3.5) | 37.1 (3.7) |  | 15.48 (3.1) | 25.0 (3.4) |
|  | OR [95%CI] | | | 1.00 (Ref) | 3.32 [1.83; 6.02]*** |  | 1.00 (Ref) | 2.23 [0.93; 5.37] |
| All figures are weighted for sampling probabilities and differential non-response. Prospective figures are adjusted for baseline status/score.  SE = standard error, OR = odds ratio, CI = confidence interval, Coeff = coefficient.  **p*<0.05, ***p*<0.01, ****p*<0.001.  Possible scores on the quality of life scale range from 0-57, and on life satisfaction scale range from 0-30. | | | | | | | | |

| **Supplementary Table 2** Unadjusted cross-sectional and prospective associations between perceived discrimination attributed to sexual orientation or any other reason and health and wellbeing outcomes | | | | | | | | | | |
| --- | --- | --- | --- | --- | --- | --- | --- | --- | --- | --- |
|  | |  |  | **Cross-sectional** | | |  | **Prospective** | | |
|  | |  |  | **No perceived discrimination** | **Perceived discrimination for any other reason** | **Perceived discrimination based on sexual orientation** |  | **No perceived discrimination** | **Perceived discrimination for any other reason** | **Perceived discrimination based on sexual orientation** |
| Fair/poor self-rated health | | | |  |  |  |  |  |  |  |
|  | % (SE) | | | 17.1 (3.5) | 27.9 (3.8) | 50.0 (10.5) |  | 26.1 (3.2) | 23.1 (3.6) | 32.5 (10.0) |
|  | OR [95%CI] | | | 1.00 (Ref) | 1.80 [0.98; 3.33] | 4.42 [1.47; 13.30]** |  | 1.00 (Ref) | 0.86 [0.38; 1.93] | 1.74 [0.36; 8.40] |
| Limiting long-standing illness | | | |  |  |  |  |  |  |  |
|  | % (SE) | | | 30.1 (3.9) | 40.2 (4.3) | 43.8 (11.9) |  | 35.1 (3.6) | 32.5 (4.0) | 51.2 (11.1) |
|  | OR [95%CI] | | | 1.00 (Ref) | 1.57 [0.92; 2.69] | 1.75 [0.59; 5.18] |  | 1.00 (Ref) | 0.81 [0.40; 1.68] | 2.56 [0.62; 10.60] |
| Depressive symptoms above threshold | | | |  |  |  |  |  |  |  |
|  | % (SE) | | | 10.5 (3.2) | 24.0 (3.4) | 50.0 (9.4) |  | 13.0 (2.7) | 12.2 (3.0) | 11.0 (8.2) |
|  | OR [95%CI] | | | 1.00 (Ref) | 2.62 [1.28; 5.39]** | 7.66 [2.41; 24.30]** |  | 1.00 (Ref) | 1.03 [0.36; 2.96] | 1.00 [0.16; 6.11] |
| Quality of life | | | |  |  |  |  |  |  |  |
|  | Mean score (SE) | | | 43.25 (0.75) | 39.80 (0.83) | 38.93 (2.41) |  | 42.96 (0.56) | 41.80 (0.61) | 39.42 (1.61) |
|  | Coeff. [95%CI] | | | Ref | -3.45 [-5.66; -1.25]** | -4.32 [-9.30; 0.65] |  | Ref | -1.16 [-2.81; 0.49] | -3.54 [-6.93; -0.16]* |
| Life satisfaction | | | |  |  |  |  |  |  |  |
|  | Mean score (SE) | | | 21.01 (0.57) | 20.24 (0.63) | 17.57 (1.82) |  | 21.74 (0.47) | 20.10 (0.52) | 20.17 (1.37) |
|  | Coeff. [95%CI] | | | Ref | -0.77 [-2.43; 0.89] | -3.44 [-7.18; 0.31] |  | Ref | -1.63 [-3.02; -0.25]* | -1.57 [-4.43; 1.30] |
| High loneliness | | | |  |  |  |  |  |  |  |
|  | % (SE) | | | 15.2 (3.5) | 33.9 (3.8) | 56.3 (10.6) |  | 14.9 (3.1) | 23.0 (3.5) | 40.5 (9.4) |
|  | OR [95%CI] | | | 1.00 (Ref) | 2.90 [1.56; 5.39]** | 6.90 [2.26; 21.11]** |  | 1.00 (Ref) | 2.05 [0.82; 5.14] | 5.38 [1.14; 25.34]* |
| All figures are weighted for sampling probabilities and differential non-response. Prospective figures are adjusted for baseline status/score.  SE = standard error, OR = odds ratio, CI = confidence interval, Coeff = coefficient.  **p*<0.05, ***p*<0.01, ****p*<0.001.  Possible scores on the quality of life scale range from 0-57, and on life satisfaction scale range from 0-30. | | | | | | | | | | |
